# Bilateral verses bilateral with tri-segmental endoscopic drainage using metal stents for high-grade malignant hilar biliary obstructions: A multicenter, randomized controlled trial: BRAVE study (BRAVE study)

**DOI:** 10.1097/MD.0000000000030857

**Published:** 2022-10-07

**Authors:** Kazuyuki Matsumoto, Toshiharu Mitsuhashi, Hirofumi Kawamoto, Etsuji Ishida, Masakuni Fujii, Yutaka Akimoto, Hiroyuki Seki, Yuki Ishihara, Taiji Ogawa, Tatsuhiro Yamazaki, Yuki Fujii, Hironari Kato

**Affiliations:** a Department of Gastroenterology and Hepatology, Okayama University Hospital, Okayama, Japan; b Center for Innovative Clinical Medicine, Okayama University Hospital, Okayama, Japan; c Department of General Internal Medicine 2, Kawasaki Medical School, Okayama, Japan; d Department of Gastroenterology and Hepatology, Kurashiki Central Hospital, Okayama, Japan; e Department of Internal Medicine, Okayama Saiseikai General Hospital, Okayama, Japan; f Department of Gastroenterology, Japanese Red Cross Okayama Hospital, Okayama, Japan; g Department of Gastroenterology, Mitoyo General Hospital, Kanonji, Japan; h Department of Gastroenterology, Iwakuni Medical Center, Hiroshima, Japan; i Department of Gastroenterology, Tsuyama Central Hospital, Okayama, Japan.

**Keywords:** bilateral drainage, bile duct obstruction, endoscopic biliary drainage, neoplasms, self-expandable metallic stents

## Abstract

Bilateral endoscopic drainage with self-expanding metallic stent (SEMS) can be used to manage hilar malignant biliary obstruction (HMBO) more effectively in comparison to unilateral drainage. An increased drainage area is predicted to prolong stent patency and patient survival. However, few reports have described the utility of trisegmental drainage and the benefits of using trisegmental drainage remain unknown. Thus, we launched a randomized clinical trial (RCT) to compare the clinical outcomes between bilateral and trisegmental drainage using SEMSs in patients with high-grade HMBO.

**Methods and analysis:**

This study was conducted as a multicenter randomized control trial (RCT) in 8 high-volume medical centers in Japan, and will prove the non-inferiority of bilateral drainage to trisegmental drainage. Patients with unresectable HMBO with Bismuth type IIIa or IV who pass the inclusion and exclusion criteria will be randomized to receive bilateral or trisegmental drainage at a 1:1 ratio. At each center, the on-site study investigators will obtain informed consent from the candidates, and will use an electronic data capture system (REDCap) to input necessary information, and register candidates with the registration secretariat. The primary endpoint is the rate of non-recurrent biliary obstruction (RBO) at 180 days after SEMSs placement. A −10% non-inferiority margin is assumed in the statistical analysis of the primary endpoint. Secondary endpoints include the rate of technical and clinical success, time to recurrent biliary obstruction (TRBO), causes of RBO, procedure-related adverse events (AEs), procedure time, TRBO with or without endoscopic sphincterotomy, overall survival, and the technical and clinical success rates at reintervention.

**Discussion:**

If the non-inferiority of bilateral drainage is demonstrated, it is predicted that the procedure time will be shortened and the medical cost will be reduced, which will be beneficial to the patient and the medical economy.

**Trial registration:**

Registered in Japan Registry of Clinical Trial-Registration (trial number. jRCTs062220038). This version number 1. Protocol dated Jun 23, 2022.

## 1. Introduction

Endoscopic management of high-grade hilar malignant biliary obstruction (HMBO) is technically challenging, and the optimal liver drainage volume remains controversial.^[1–5]^ Vienne et al^[1]^ reported that drainage of ≥50% of the total liver volume was associated with the achievement of effective drainage and an improved prognosis. Theoretically, bilateral or unilateral drainage should be selected to obtain ≥50% drainage. However, the liver function in the drainage area does not coincide with the calculated liver volume. This is because some patients with high-grade HMBO have portal vein infiltration and/or highly divided bile duct branches at the drainage area due to the presence of tumors. Thus, in certain patients, sufficient drainage cannot be obtained using unilateral drainage alone. Regarding bilateral and unilateral drainage, a randomized control trail (RCT) on unilateral and bilateral drainage performed using self-expandable metallic stent (SEMS) in patients with advanced HMBO showed that bilateral drainage was associated with superior stent patency, lower reintervention rates, and similar adverse event rates in comparison to unilateral drainage.^[6]^ Accordingly, the results of the aforementioned study predicted that a smaller non-drainage area was associated with fewer obstruction events (e.g., cholangitis and longer patency). However, it remains unclear whether the use of bilateral drainage involving the left and right lobes (anterior and posterior) would promote better outcomes for patients with high-grade HMBO.^[6–9]^

We previously launched a multicenter retrospective study comparing stent patency between bilateral (the left hepatic duct [LDH], the anterior branch of the right hepatic duct [a-RHD] or posterior branch of the right hepatic duct [p-RHD]) and trisegmental (the LDH, a-RHD and p-RHD) drainage using uncovered SEMSs in patients with high-grade HMBO (Bismuth Type IIIa and IV).^[9]^ The technical success rates of the bilateral and trisegmental drainage groups were 95% (34/36) and 90% (80/89), respectively (were 95% (34/36) and 90% (80/89) 0.41). Contrary to our expectations, there was no significant difference in stent patency between bilateral and trisegmental drainage (Median 226 vs 170 days, *P* = .34, long-rank test). Moreover, the trisegmental group had a significantly longer procedure time in comparison to the bilateral group (78 min vs 54 min, respectively; *P* = .0022). The trisegmental drainage method is indeed technically difficult and associated with higher medical costs in comparison to bilateral drainage. Although there were many limitations in the past study, its benefits and complications remain unclear.

The purpose of this RCT is to compare the clinical outcomes between bilateral and trisegmental drainage in a patient with high-grade HMBO. Based on past data, this design will prove the non-inferiority of bilateral drainage to trisegmental drainage in terms of recurrent biliary obstruction (RBO). If the non-inferiority of bilateral drainage is demonstrated, it is predicted that the procedure time will be shortened, and the medical cost will be reduced, which will be beneficial to the patient and the medical economy.

## 2. Methods and analysis

### 2.1. Ethics approval and patient consent

This research adheres to the principles of the declaration of Helsinki. Written informed consent from all screened patients will be obtained before the procedures start. The study protocol has been approved by Okayama University Certified Review Board (approval number. CRB22-002) and was registered in Japan Registry of Clinical Trial-Registration (trial number. jRCTs062220038) on June 23, 2022.

### 2.2. Study design

**Figure 1.**
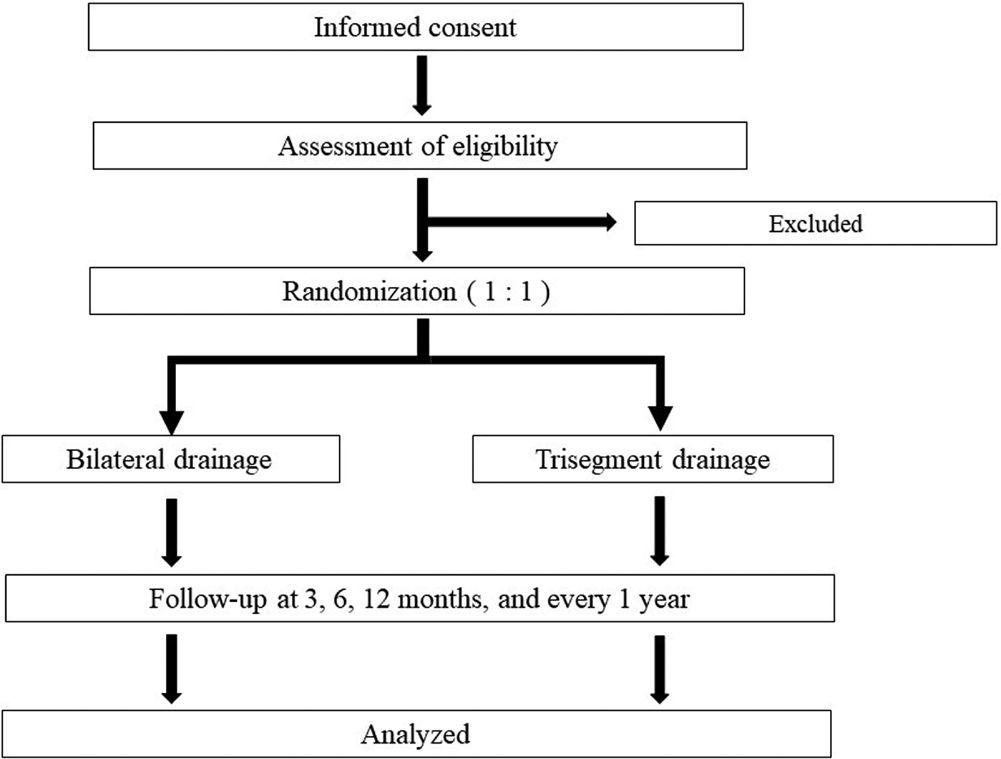
A flow chart of the study design.

**Figure 2.**
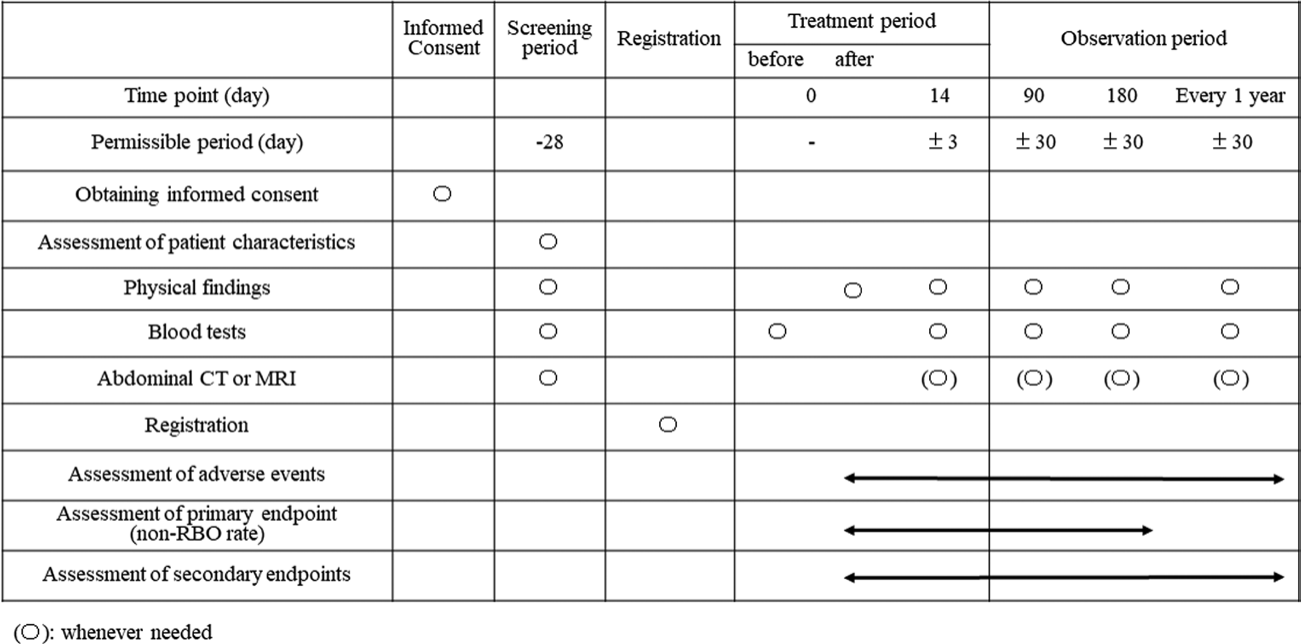
Schedule for data collection. RBO = recurrent biliary obstruction.

This study is conducted as a multicenter prospective RCT in 8 high-volume medical centers in Japan, and will prove the non-inferiority of bilateral drainage to trisegmental drainage in terms of RBO in patients with high-grade HMBO. At each center, the on-site study investigators will obtain informed consent from candidates, and they will use an electronic data capture system (REDCap) to input necessary information, confirm that the candidates meet the eligibility criteria, and register the candidates with the registration secretariat. After confirming that a candidate meets the criteria, a registration number will be issued, and the registration will be considered complete. Patients who have completed registration will be randomly assigned (1:1) to receive endoscopic bilateral or trisegmental drainage to treat biliary obstruction. Patients will be followed up until death or 6 months after the final registration of this study. An overview of the protocol is shown in Figure 1 and the schedule for enrollment, interventions, and assessment is shown in Figure 2.

### 2.3. Patient eligibility

The following inclusion and exclusion criteria will be applied:

### 2.4. Inclusion criteria

1. Unresectable HMBO malignant hilar biliary obstruction with Bismuth type IIIa or IV.
2. Age >18 years.
3. Patient with fully informed consent.

### 2.5. Exclusion criteria

1. Reconstruction of bowel with procedure other than Billroth 1.
2. Inferior border of the biliary stricture extends close to the duodenal papilla necessitating stenting across the papilla of Vater.
3. Endoscopic drainage will be ineffective due to severely divided bile duct, or severe portal vein invasion due to multiple liver metastasis.
4. Radical resection, radiation therapy, and proton/particle therapy will be planned.
5. Patient is pregnant or possibly pregnant.
6. Patient is not expected to live for ≥1 month.
7. Patient judged by the investigator to be inappropriate for enrollment.

### 2.6. Endpoints and definition

#### 2.6.1. Primary endpoint

The primary endpoint is the non-RBO rate at 180 days after stent insertion. The non-RBO rate is defined as the percentage of patients who do not experience RBO from the date of stent insertion to 180 days after treatment. Patients who are lost follow-up or die without RBO within 180 days of treatment are classified as non-RBO. RBO is defined as a composite endpoint of either occlusion or migration, according to the 2014 Tokyo criteria.^[10]^ Stent occlusion is defined as present when there is biochemical evidence of cholestasis (i.e. elevated liver enzymes in comparison to baseline values), accompanied by biliary dilation on imaging studies or endoscopic findings suggesting biliary dilation. Stent migration is diagnosed when reintervention reveals a completely or partially migrated SEMS as a cause of RBO.

#### 2.6.2. Secondary endpoints

The secondary endpoints are as follows:

1. Technical success rate: Technical success is defined as successful stent placement in each planned drainage area. For patients randomized to the trisegmental drainage group, technical success is defined as the successful placement of three SEMSs in the planned drainage area. For patients randomized to the bilateral drainage group, technical success is defined as the successful placement of two SEMSs in the planned drainage area.
2. Clinical success rate: Clinical success is defined as a 50% decrease in or normalization of the patient’s bilirubin level within 14 days of stent placement.
3. Time to recurrent biliary obstruction (TRBO): TRBO is defined as the period between the SEMS deployment and RBO. Data regarding patients who are lost to follow-up or who die without RBO will be censored at the last observation date.
4. Causes of RBO. The causes of SEMS occlusion can be categorized as follows: tumor ingrowth/mucosal hyperplasia; tumor overgrowth; sludge with/without stones; hemobilia; food impaction; bile duct kinking; and other.
5. Procedure-related AE: Early (within 30 days after treatment) and late (≥31 days) adverse events (AEs) include post-procedural pancreatitis, cholecystitis, non-occlusion cholangitis, perforation, bleeding with scope, desaturation of oxygen, aspiration pneumonia, etc., according the 2014 Tokyo criteria.^[10]^
6. Procedure time: Procedure time is defined as the time from biliary cannulation to scope removal.
7. Comparison of TRBO between patients with endoscopic sphincterotomy and without endoscopic sphincterotomy.
8. Overall survival time: Overall survival time is measured from the day the SEMSs are deployed for HMBO to the date of death or loss to follow-up.
9. Technical and clinical success rates at reintervention: Technical success at reintervention is defined as successful PS deployment in all initially placed SEMSs. Definition of clinical success at reintervention is defined the same as clinical success after initial SEMS placement.

#### 2.6.3. Randomization and blinding

Subjects will be assigned to one of the treatment methods at a ratio of 1:1 by dynamic allocation according to a web-based registration program system (REDCap) based on the baseline factors for treatment allocation, including the type of biliary obstruction (Bismuth type IIIa or IV) and chemotherapy (planned or not). The registration secretariat will strictly control the program to prevent leakage of the assignment information to other involved personnel. Blinding will not be used in this study.

### 2.7. Study procedure

For effective drainage of the liver volume, atrophic liver and dominant right or left portal vein occlusion sites with or without liver atrophy will be avoided as much as possible. While waiting for pathological confirmation and a decision on operability, temporary biliary decompression by percutaneous drainage, nasobiliary catheters or plastic stents (PSs) is allowed until 8 weeks.

All patients will undergo ERCP using a standard duodenal scope (TJF-290, TJF-260, or JF-260V; Olympus Optical Co, Ltd., Tokyo, Japan). The procedure will be performed with the patient in a prone or semi-prone position under conscious sedation using intravenous anesthetic in the endoscopy room. This study will use the Zeo stent V (Zeon Medical Inc., Tokyo, Japan). In bilateral drainage, two SEMSs will be placed at the LHD and the a-RHD or p-RHD depending on the liver volume on CT. In trisegmental drainage three SEMSs will be placed at the LHD, a-RHD, and p-RHD. In all patients stent deployment will be performed with the partial stent-in-stent (PSIS) method using SEMSs of 10 mm in diameter. The lengths of SEMSs were 4, 6, 8, and 10 cm, depending on the length of the stricture. Endoscopic sphincterotomy will be performed depending on the operator, and all SEMSs will be placed above the papilla.

For reintervention, after confirming the cause of RBO, 6-7F PSs or SEMSs (company not specified) are inserted into each lumen of the previously deployed SEMS.

### 2.8. Follow-up

Follow-up examinations are scheduled for at least 3, 6, 12 months and every 1 year to evaluate the general condition of the patient, and perform blood testing. Patients who receive chemotherapy will be followed-up basically every 1 to 2 weeks. The study will end 6 months after the registration of the last patient. If the patient is unable to attend hospital, follow-up will be conducted over the phone. If follow-up information cannot be obtained, the patient will be considered ne lost to follow-up.

### 2.9. Sample size calculation

The primary endpoint of this study is the stent non-RBO rate. According to the results of our past study,^[9]^ the non-RBO rate of the bilateral drainage group was 72.3% with a 95% confidence interval: CI 55.8% to 84.9%. The non-RBO rate of the trisegmental drainage group was 52.9% with a 95% CI 42.4% to 63.2%. Based on this, the expected value of the non-RBO rate of bilateral drainage group is set at 72.3%, and that of trisegmental drainage group is set at 52.9%. We set the non-inferiority margin as 10%. Assuming testing with a one-sided α value of 0.05 using the exact method assuming a binomial distribution under the above set values, 33 cases are required for detection with a statistical power of 80%. Considering the exclusion/dropout rate of about 15%, the target number of cases is set at 76 cases.

### 2.10. Statistical analysis

The full analysis set (FAS) was defined as all randomized cases excluding minimally excludable cases. Minimally excludable cases were defined as follows: Ineligible subjects who were incorrectly enrolled, cases in which the drainage treatment itself was not performed after allocation, cases in which all post-assignment outcome data are missing, and cases in which consent is withdrawn. The population of the FAS who complete treatment is defined as the per protocol set (PPS). This includes cases in which the treatment complies with the allocation, without missing data, and without serious protocol violations.

Intention-to-treat (ITT) and per-protocol (PP) methods are used for the analysis. The ITT analysis is the main analysis based on the FAS, whereas the PP analysis is a sub-analysis based on the PPS. Demographic and clinical characteristics will be presented as the median (interquartile range) for continuous variables and frequency (percentage) for categorical variables. Welch’s *t* test and a χ^2^ test will be used to compare continuous and categorical variables, respectively.

The primary endpoint analysis will be performed according to the ITT principle. A point estimate of the difference between the non-RBO proportion in the bilateral drainage group and the trisegmental drainage group and its 95% Wald confidence interval will be calculated. Non-inferiority is considered statistically significant when the lower limit of 95% CI exceeds the non-inferiority margin of 10%, or when the *P* value is less than 5%.

Secondary endpoint analyses will be conducted to provide supplemental discussion to the results of the primary endpoint analysis. These analyses are exploratory and will not be adjusted for multiplicity, and will be conducted without considering the margin of non-inferiority. For the secondary endpoints indicated by proportions, point estimates and 95% Wald CIs of the difference between groups will be calculated. For the continuous secondary endpoints, point estimates and 95% CIs of the mean difference between groups will be calculated. Secondary endpoints indicated by categorical variables will be enumerated for each group and compared by a χ^2^ test if necessary. The survival time analysis for TRBO and overall survival will be estimated by the Kaplan–Meier method and compared by a log-rank test. Hazard ratios and their 95% Wald CIs will be obtained using the Cox proportional hazards model. The significance level is 5%, and Stata version 17 (Stata Corp. College Station, Texas) will be used to perform all statistical analyses.

### 2.11. Adverse event reporting

The principal investigator of the institution where the serious AE occurs should take appropriate measures, regardless of whether or not there is a causal relationship with the research, and should immediately report the details of the event to the head of the research institution and the principal investigator, in accordance with the regulations of the respective medical institution. A serious adverse event is an AE occurring during the procedure or any time after the procedure that fulfills ≥ of the following criteria:

1. Results in death or is immediately life-threatening.
2. Requires in-patient hospitalization or prolongation of existing hospitalization (≥10 days).
3. Results in persistent or significant disability or incapacity.
4. A congenital abnormality or birth defect.

### 2.12. Monitoring

Off-site monitoring (using e-mail, web meeting tool, electronic data capture system) will be carried out 30% of enrolled patients throughout the trial by an independent data monitoring unit. The monitoring unit will collect information on the status of accumulation, inclusion/ exclusion criteria, serious AEs, and any other relevant information, and strive to provide feedback to participating institutions for early resolution if there are any problems. The monitoring committee will also report any serious AEs to the committee for efficacy and safety assessment. Data quality check will be performed using an electronic data capture system every 6 months by an independent unit. Auditing will not be performed in this study.

## 3. Discussion

Bilateral endoscopic drainage with SEMS can be used to effectively manage patients with HMBO.^[1–5]^ However, few reports have described the utility of trisegmental drainage and the benefits of using trisegmental drainage remain unknown.^[6–9]^ There have been no RCTs to compare bilateral and trisegmental drainage in patients with high-grade HMBO. The primary endpoint of this multicenter RCT is the comparison of the non-RBO rate at 6 months between bilateral and trisegmental drainage in patient with high-grade HMBO. The secondary endpoints are the rates of technical and clinical success, procedure time, procedure related AEs, TRBO, technical and the clinically successful reintervention, and overall survival in the two groups. If the non-inferiority of bilateral drainage is demonstrated, it is predicted that the procedure time will be shortened, and medical costs will be reduced.

Recent studies have reported that the technical success rate and stent patency in patients with HMBO treated with bilateral drainage using SEMS were 90% to 100% and 4.2 to 16.3 months, respectively.^[2–5]^ Only three previous studies have been published on trisegmental drainage using three SEMSs; these reported technical success rates and stent patency of 82% to 100% and 5.7 to 7.1 months, respectively.^[6–9]^ Each study was associated with some limitations, the benefit of trisegmental drainage is not clear. In our previous study, we compared stent patency between bilateral and trisegmental drainage in patients with HMBO limited to Bismuth IIIa or IV; however, the study was associated with some limitations.^[9]^ First, this was a retrospective study that included a small number of patients in the bilateral drainage group. Second, the decision to perform bilateral or trisegmental drainage was left to the discretion of each physician. Third, although most cases underwent drainage using the PSIS method, three drainage methods were utilized. Finally, various devices, SEMSs, and PSs were used.

This study will resolve the limitations of previous studies. We believe that this RCT will be able to correctly assess the study endpoints.

## Author contributions

**Conceptualization:** Kazuyuki Matsumoto, Hironari Kato.

**Data curation:** Tatsuhiro Yamazaki, Yuki Fujii.

**Formal analysis:** Toshiharu Mitsuhashi.

**Funding acquisition:** Kazuyuki Matsumoto.

**Investigation:** Hirofumi Kawamoto, Etsuji Ishida, Masakuni Fujii, Yutaka Akimoto, Hiroyuki Seki, Yuki Ishihara, Taiji Ogawa.

**Methodology:** Kazuyuki Matsumoto, Toshiharu Mitsuhashi.

**Writing – original draft:** Kazuyuki Matsumoto, Toshiharu Mitsuhashi.

**Writing – review & editing:** Hironari Kato.
